# Supplementary material for: Assessing private provider perceptions and the acceptability of video observed treatment technology for tuberculosis treatment adherence in three cities across Viet Nam
Source: PLoS One. 2021 May 7;16(5):e0250644. doi: 10.1371/journal.pone.0250644 (PMC8104441; doi:10.1371/journal.pone.0250644)
Supplement: S1 Table — (PDF) [file pone.0250644.s001.pdf]

S1 Table: Cronbach's alpha (reliability coefficient)

Constructs of healthcare intervention acceptability and their components	Item-test correlation	Item-rest correlation	Average interitem covariance	Cronbach's alpha
**Ethicality**[a]				
Belief that observation is best strategy for adherence	0.588	0.510	0.214	**0.865**
Willingness to test new approaches	0.436	0.362	0.227	**0.871**
**Intervention Coherence**[a]				
Identify side effects faster	0.646	0.570	0.209	**0.863**
Identify people at risk of stopping treatment faster	0.594	0.536	0.220	**0.865**
**Burden**[b]				
Time requirement from doctor	0.632	0.551	0.209	**0.864**
Time requirement from patient	0.616	0.547	0.214	**0.864**
**Opportunity Cost**[a]				
Save time for doctor	0.717	0.655	0.205	**0.859**
Save money for doctor	0.557	0.473	0.216	**0.867**
**Perceived Effectiveness**[a]				
Help in providing differentiated care	0.647	0.585	0.214	**0.863**
Help patients adhere to treatment	0.592	0.528	0.218	**0.865**
**Self-Efficacy**[a]				
Confidence monitoring treatment through VOT	0.586	0.516	0.217	**0.865**
Confidence providing differentiated care through VOT	0.565	0.504	0.221	**0.866**
**Affective Attitude**[a]				
Addresses problems which patients face	0.553	0.472	0.217	**0.867**
Be beneficial for doctor's practice and patients	0.674	0.615	0.212	**0.861**
Be relevant for all of doctor's TB patients	0.562	0.493	0.219	**0.866**
**Implementation/Usability**[a]				
Doctor's concerns about patient confidentiality	0.377	0.260	0.227	**0.879**
Doctor's comfort with receiving support from study staff	0.489	0.413	0.224	**0.869**
**Total**			0.217	**0.873**

[a]: 1 (Strongly Disagree) to 5 (Strong Agree)

[b]: 1 (Very Difficult) to 5 (Very Easy)
