# Supplementary material for: Assessing private provider perceptions and the acceptability of video observed treatment technology for tuberculosis treatment adherence in three cities across Viet Nam
Source: PLoS One. 2021 May 7;16(5):e0250644. doi: 10.1371/journal.pone.0250644 (PMC8104441; doi:10.1371/journal.pone.0250644)
Supplement: S2 Table — (PDF) [file pone.0250644.s002.pdf]

S2 Table: Constructs of acceptability associated with the willingness to use VOT in Ha Noi

Constructs of healthcare intervention acceptability and their components	Would use VOT (N= 15)		Would not use VOT (N=2)		Mann-Whitney *U* test
	Median (IQR)	Mean (95%CI)	Median (IQR)	Mean (95%CI)	
**Ethicality[a]**					
Doctor's belief that observation is best strategy for adherence	4 (4-5)	4.5 (4.2-4.7)	3.5 (3-4)	3.5 (2.4-4.6)	0.060
Doctor's willingness to test new approaches	4 (4-5)	4.3 (3.9-4.6)	2.5 (2-3)	2.5 (1.4-3.6)	**0.018**
**Intervention Coherence[a]**					
VOT would help identify side effects faster	4 (3-4)	3.6 (3.1-4.1)	2 (2-2)	2.0 (2-0-2.0)	**0.042**
VOT would help identify people at risk of stopping treatment faster	4 (4-4)	4.0 (3.6-4.4)	4 (4-4)	4.0 (4.0-4.0)	0.853
**Burden[b]**					
VOT time requirement from doctor	4 (3-4)	3.5 (3.1-3.9)	2 (2-2)	2.0 (2.0-2.0)	**0.026**
VOT time requirement from patient	3 (2-4)	3.2 (2.7-3.7)	2.5 (2-3)	2.5 (1.4-3.6)	0.267
**Opportunity Cost[a]**					
VOT would save time for doctor	4 (3-4)	3.7 (3.3-4.2)	3 (2-4)	3.0 (0.9-5.1)	0.371
VOT would save money for doctor	3 (2-4)	3.2 (2.7-3.7)	2 (2-2)	2.0 (2.0-2.0)	0.085
**Perceived Effectiveness[a]**					
VOT would help in providing differentiated care	4 (4-4)	3.9 (3.5-4.3)	2 (2-2)	2.0 (2.0-2.0)	**0.014**
VOT would help patients adhere to treatment	4 (4-4)	4.1 (3.9-4.4)	3 (2-4)	3.0 (0.9-5.1)	0.115
**Self-Efficacy[a]**					
Doctor's confidence in ability to monitor treatment through VOT	4 (3-4)	3.7 (3.3-4.1)	2.5 (2-3)	2.5 (1.4-3.6)	**0.043**
Doctor's confidence in ability to provide differentiated care through VOT	4 (4-4)	3.8 (3.4-4.2)	2 (2-2)	2.0 (2.0-2.0)	**0.014**
**Affective Attitude[a]**					
Addresses problems which patients face	4 (3-4)	3.7 (3.4-4.1)	3.5 (3-4)	3.5 (2.4-4.6)	0.538
Be beneficial for doctor's practice and patients	4 (4-4)	3.9 (3.5-4.3)	3.5 (3-4)	3.5 (2.4-4.6)	0.266
Be relevant for all of doctor's TB patients	2 (2-3)	2.5 (2.1-2.9)	2 (2-2)	2.0 (2.0-2.0)	0.320
**Implementation/Usability[a]**					
Doctor's concerns about patient confidentiality	3 (2-4)	2.9 (2.4-3.5)	3 (2-4)	3.0 (0.9-5.1)	0.935
Doctor's comfort with receiving support from study staff	4 (4-4)	3.9 (3.6-4.3)	4 (4-4)	4.0 (4.0-4.0)	0.853

[a]: 1 (Strongly Disagree) to 5 (Strong Agree)

[b]: 1 (Very difficult) to 5 (Very easy)
