# Supplementary material for: Assessing private provider perceptions and the acceptability of video observed treatment technology for tuberculosis treatment adherence in three cities across Viet Nam
Source: PLoS One. 2021 May 7;16(5):e0250644. doi: 10.1371/journal.pone.0250644 (PMC8104441; doi:10.1371/journal.pone.0250644)
Supplement: S3 Table — (PDF) [file pone.0250644.s003.pdf]

S3 Table: Constructs of acceptability associated with the willingness to use VOT in HCMC

Constructs of healthcare intervention acceptability and their components	Would use VOT (n= 19)		Would not use VOT (n=15)		Mann-Whitney *U* test
	Median (IQR)	Mean (95%CI)	Median (IQR)	Mean (95%CI)	
**Ethicality[a]**					
Belief that observation is best strategy for adherence	4 (3-4)	3.7 (3.1-4.2)	4 (2-4)	3.5 (2.9-4.0)	0.416
Willingness to test new approaches	4 (4-4)	3.8 (3.4-4.2)	4 (3-4)	3.7 (3.3-4.1)	0.475
**Intervention Coherence[a]**					
Identify side effects faster	4 (3-4)	3.5 (3.1-3.9)	2 (2-4)	2.7 (2.2-3.3)	**0.032**
Identify people at risk of stopping treatment faster	4 (3-4)	3.6 (3.3-3.9)	4 (3-4)	3.5 (3.1-3.9)	0.883
**Burden[b]**					
Time requirement from doctor	2 (1-3)	2.3 (1.8-2.8)	2 (2-3)	2.5 (2.0-2.9)	0.592
Time requirement from patient	3 (2-3)	2.7 (2.4-3.1)	2 (2-3)	2.3 (1.9-2.8)	0.170
**Opportunity Cost[a]**					
Save time for doctor	4 (2 -4)	3.5 (3.0-3.9)	2 (2-4)	2.7 (2.2-3.2)	**0.035**
Save money for doctor	2 (2-4)	2.8 (2.3-3.2)	2 (2-2)	2.1 (1.8-2.4)	**0.024**
**Perceived Effectiveness[a]**					
Help in providing differentiated care	4 (3-4)	3.7 (3.4-4.0)	4 (2-4)	3.3 (2.8-3.7)	0.146
Help patients adhere to treatment	4 (4-4)	3.7 (3.4-4.1)	4 (3-4)	3.5 (3.1-4.0)	0.474
**Self-Efficacy[a]**					
Confidence monitoring treatment through VOT	4 (3-4)	3.4 (3.0-3.8)	2 (2-4)	2.7 (2.2-3.1)	**0.028**
Confidence providing differentiated care through VOT	4 (4-4)	3.9 (3.8-4.0)	4 (2-4)	3.1 (2.6-3.6)	**0.004**
**Affective Attitude[a]**					
Addresses problems which patients face	4 (3-4)	3.6 (3.3-3.9)	2 (2-4)	2.7 (2.2-3.1)	**0.004**
Be beneficial for doctor's practice and patients	4 (3-4)	3.5 (3.2-3.9)	2 (2-4)	2.8 (2.3-3.3)	**0.023**
Be relevant for all of doctor's TB patients	2 (2-2)	2.4 (2.0-2.7)	2 (2-2)	2.0 (1.8-2.2)	0.123
**Implementation/Usability[a]**					
Doctor's concerns about patient confidentiality	4 (4-4)	3.8 (3.4-4.2)	4 (2-4)	3.3 (2.7-3.9)	0.270
Doctor's comfort with receiving support from study staff	4 (4-4)	3.9 (3.6-4.2)	4 (2-4)	3.2 (2.8-3.8)	**0.044**

[a]: 1 (Strongly disagree) to 5 (Strongly agree)

[b]: 1 (Very difficult) to 5 (Very easy)
