# Supplementary material for: Assessing private provider perceptions and the acceptability of video observed treatment technology for tuberculosis treatment adherence in three cities across Viet Nam
Source: PLoS One. 2021 May 7;16(5):e0250644. doi: 10.1371/journal.pone.0250644 (PMC8104441; doi:10.1371/journal.pone.0250644)
Supplement: S4 Table — (PDF) [file pone.0250644.s004.pdf]

S4 Table: Comparison of VOT acceptability among private-sector doctors for Ho Chi Minh City and Ha Noi

Constructs of healthcare intervention acceptability and their components	Would use VOT (N= 34)		Would not use VOT (N=17)		Mann-Whitney *U* test
	Median (IQR)	Mean (95%CI)	Median (IQR)	Mean (95%CI)	
**Ethicality**[a]					
Doctor's belief that observation is best strategy for adherence	4 (4-5)	4.0 (3.7-4.4)	4 (3-4)	3.5 (3.0-3.9)	**0.025**
Doctor's willingness to test new approaches	4 (4-4)	4.0 (3.8-4.3)	4 (3-4)	3.6 (3.2-4.0)	**0.041**
**Intervention Coherence**[a]					
VOT would help identify side effects faster	4 (3-4)	3.5 (3.2-3.8)	2 (2-4)	2.6 (2.2-3.1)	**0.004**
VOT would help identify people at risk of stopping treatment faster	4 (4-4)	3.8 (3.5-4.0)	4 (3-4)	3.6 (3.2-3.9)	0.387
**Burden**[b]					
VOT time requirement from doctor	3 (2-4)	2.8 (2.5-3.2)	2 (2-3)	2.4 (2.0-2.8)	0.144
VOT time requirement from patient	3 (2-4)	2.9 (2.6-3.2)	2 (2-3)	2.3 (1.9-2.8)	0.031
**Opportunity Cost**[a]					
VOT would save time for doctor	4 (3 -4)	3.6 (3.3-3.9)	2 (2-4)	2.8 (2.3-3.2)	**0.007**
VOT would save money for doctor	3 (2-4)	3.0 (2.6-3.3)	2 (2-2)	2.1 (1.9-2.3)	**0.001**
**Perceived Effectiveness**[a]					
VOT would help in providing differentiated care	4 (4-4)	3.8 (3.6-4.0)	3 (2-4)	3.1 (2.7-3.6)	**0.008**
VOT would help patients adhere to treatment	4 (4-4)	3.9 (3.7-4.2)	4 (3-4)	3.5 (3.0-3.9)	0.067
**Self-Efficacy**[a]					
Doctor's confidence in ability to monitor treatment through VOT	4 (3-4)	3.5 (3.3-3.8)	2 (2-3)	2.6 (2.2-3.1)	**0.001**
Doctor's confidence in ability to provide differentiated care through VOT	4 (4-4)	3.9 (3.7-4.0)	3 (2-4)	3.0 (2.5-3.5)	**0.001**
**Affective Attitude**[a]					
Addresses problems which patients face	4 (3-4)	3.6 (3.4-3.9)	2 (2-4)	2.8 (2.3-3.2)	**0.001**
Be beneficial for doctor's practice and patients	4 (4-4)	3.7 (3.4-4.0)	3 (2-4)	2.9 (2.4-3.4)	**0.003**
Be relevant for all of doctor's TB patients	2 (2-3)	2.4 (2.2-2.7)	2 (2-2)	2.0 (1.8-2.2)	**0.036**
**Implementation/Usability**[a]					
Doctor's concerns about patient confidentiality	4 (2-4)	3.4 (3.1-3.8)	4 (2-4)	3.3 (2.7-3.9)	0.776
Doctor's comfort with receiving support from study staff	4 (4-4)	3.9 (3.7-4.1)	4 (2-4)	3.3 (2.9-3.8)	**0.039**

[a]: 1 (Strongly Disagree) to 5 (Strong Agree)

[b]: 1 (Very Difficult) to 5 (Very Easy)
