# Supplementary material for: Assessing private provider perceptions and the acceptability of video observed treatment technology for tuberculosis treatment adherence in three cities across Viet Nam
Source: PLoS One. 2021 May 7;16(5):e0250644. doi: 10.1371/journal.pone.0250644 (PMC8104441; doi:10.1371/journal.pone.0250644)
Supplement: S5 Table — (PDF) [file pone.0250644.s005.pdf]

S5 Table: Constructs of acceptability associated with the willingness to use VOT in Ha Noi and Hai Phong

Constructs of healthcare intervention acceptability and their components	Would use VOT (n= 43)		Would not use VOT (n=2)		Mann-Whitney *U* test
	Median (IQR)	Mean (95%CI)	Median (IQR)	Mean (95%CI)	
**Ethicality[a]**					
Belief that observation is best strategy for adherence	5 (4-5)	4.5 (4.4-4.7)	3.5 (3-4)	3.5 (2.5-4.5)	**0.041**
Willingness to test new approaches	4 (4-5)	4.3 (4.1-4.4)	2.5 (2-3)	2.5 (1.5-3.5)	**0.005**
**Intervention Coherence[a]**					
Identify side effects faster	4 (3-4)	3.7 (3.4-4.0)	2 (2-2)	2.0 (2.0-2.0)	**0.021**
Identify people at risk of stopping treatment faster	4 (4-4)	4.0 (3.8-4.2)	4 (4-4)	4.0 (4.0-4.0)	0.869
**Burden[b]**					
Time requirement from doctor	4 (3-4)	3.6 (3.4-3.8)	2 (2-2)	2.0 (2.0-2.0)	**0.014**
Time requirement from patient	3 (3-4)	3.2 (3.0-3.4)	2.5 (2-3)	2.5 (1.5-3.5)	0.215
**Opportunity Cost[a]**					
Save time for doctor	4 (4-4)	3.9 (3.6-4.1)	3 (2-4)	3.0 (1.0-5.0)	0.202
Save money for doctor	3 (3-4)	3.3 (3.0-3.6)	2 (2-2)	2.0 (2.0-2.0)	**0.047**
**Perceived Effectiveness[a]**					
Help in providing differentiated care	4 (4-4)	4.0 (3.7-4.2)	2 (2-2)	2.0 (2.0-20)	**0.008**
Help patients adhere to treatment	4 (4-4)	4.0 (3.8-4.2)	3 (2-2)	3.0 (3.8-5.0)	0.136
**Self-Efficacy[a]**					
Confidence monitoring treatment through VOT	4 (4-4)	3.8 (3.6-4.0)	2.5 (2-3)	2.5 (1.5-3.5)	**0.012**
Confidence providing differentiated care through VOT	4 (4-4)	3.8 (3.6-4.0)	2 (2-2)	2.0 (2.0-2.0)	**0.002**
**Affective Attitude[a]**					
Addresses problems which patients face	4 (4-4)	3.9 (3.7-4.1)	3.5 (3-4)	3.5 (2.5-4.5)	0.270
Be beneficial for doctor's practice and patients	4 (4-4)	4.0 (3.8-4.2)	3.5 (3-4)	3.5 (2.5-4.5)	0.139
Be relevant for all of doctor's TB patients	3 (2-3)	2.8 (2.6-3.1)	2 (2-2)	2.0 (2.0-2.0)	0.127
**Implementation/Usability[a]**					
Doctor's concerns about patient confidentiality	2 (2-4)	2.8 (2.5-3.1)	3 (2-4)	3.0 (1.0-5.0)	0.778
Doctor's comfort with receiving support from study staff	4 (4-4)	4.0 (3.7-4.2)	4 (4-4)	4.0 (4.0-4.0)	0.942

[a]: 1 (Strongly Disagree) to 5 (Strongly Agree)

[b]: 1 (Very Difficult) to 5 (Very Easy)
