# Supplementary material for: Assessing private provider perceptions and the acceptability of video observed treatment technology for tuberculosis treatment adherence in three cities across Viet Nam
Source: PLoS One. 2021 May 7;16(5):e0250644. doi: 10.1371/journal.pone.0250644 (PMC8104441; doi:10.1371/journal.pone.0250644)
Supplement: S6 Table — (PDF) [file pone.0250644.s006.pdf]

S8 Table: Adjusting for multiple comparisons using the Holm-Bonferroni sequential correction

	P-value	adjusted α using the Holm-Bonferroni correction
**Characteristics of private providers in the survey stratified by city**		
**Sex**		
Hai Phong vs Ha Noi	0.4200	0.0500
Ha Noi vs HCMC	**0.0030**	0.0167
Hai Phong vs HCMC	**0.0140**	0.0250
**Age**		
Hai Phong vs Ha Noi	0.6580	
Ha Noi vs HCMC	0.0820	0.0250
Hai Phong vs HCMC	**0.0050**	0.0167
**Overall attitudes of private providers towards VOT**		
**Belief that observation is best strategy for adherence**		
Hai Phong vs Ha Noi	0.3780	
Ha Noi vs HCMC	0.0810	0.0250
Hai Phong vs HCMC	**0.0020**	0.0167
**Willingness to test new approaches**		
Hai Phong vs Ha Noi	0.0480	
Ha Noi vs HCMC	0.8610	0.0250
Hai Phong vs HCMC	**0.0170**	0.0167
**Identify people at risk of stopping treatment faster**		
Hai Phong vs Ha Noi	0.4670	
Ha Noi vs HCMC	0.3300	0.0250
Hai Phong vs HCMC	**0.0060**	0.0167
**Time requirement from doctor**		
Hai Phong vs Ha Noi	0.4170	0.0500
Ha Noi vs HCMC	**0.0070**	0.0250
Hai Phong vs HCMC	**0.0000**	0.0167
**Time requirement from patients**		
Hai Phong vs Ha Noi	0.4760	
Ha Noi vs HCMC	0.1230	0.0250
Hai Phong vs HCMC	**0.0090**	0.0167
**Save time for doctor**		
Hai Phong vs Ha Noi	**0.0180**	0.0250
Ha Noi vs HCMC	**0.0410**	0.0500
Hai Phong vs HCMC	**0.0010**	0.0167
**Save money for doctor**		

Hai Phong vs Ha Noi	0.3950	0.0500
Ha Noi vs HCMC	**0.0060**	0.0250
Hai Phong vs HCMC	**0.0000**	0.0167
**Confidence in ability to monitor treatment through VOT**		
Hai Phong vs Ha Noi	0.2250	
Ha Noi vs HCMC	0.1460	0.0250
Hai Phong vs HCMC	**0.0000**	0.0167
**Addresses problems which patients face**		
Hai Phong vs Ha Noi	0.3060	
Ha Noi vs HCMC	0.0910	0.0250
Hai Phong vs HCMC	**0.0030**	0.0167
**Beneficial for doctor's practice and patients**		
Hai Phong vs Ha Noi	0.4070	
Ha Noi vs HCMC	0.0540	0.0250
Hai Phong vs HCMC	**0.0000**	0.0167
**Relevant for all of doctor's TB patients**		
Hai Phong vs Ha Noi	0.0590	
Ha Noi vs HCMC	0.0320	0.0250
Hai Phong vs HCMC	**0.0000**	0.0167
**Concerns about patient confidentiality**		
Hai Phong vs Ha Noi	0.3980	
Ha Noi vs HCMC	0.1030	0.0250
Hai Phong vs HCMC	**0.0050**	0.0167
**Provider willingness to use VOT**		
Hai Phong vs Ha Noi	0.1370	0.0500
Ha Noi vs HCMC	**0.0210**	0.0250
Hai Phong vs HCMC	**0.0000**	0.0167
