# Supplementary material for: Assessing private provider perceptions and the acceptability of video observed treatment technology for tuberculosis treatment adherence in three cities across Viet Nam
Source: PLoS One. 2021 May 7;16(5):e0250644. doi: 10.1371/journal.pone.0250644 (PMC8104441; doi:10.1371/journal.pone.0250644)
Supplement: S1 Survey — (PDF) [file pone.0250644.s008.pdf]

Private Provider Acceptability Survey in English

Date of survey: __ __ - __ __ - __ __ __ __ **Name of interviewer:** ________________________________

Name of doctor: ______________________________ **Gender:** [ ] M [ ] F **District/ward:** ______________

Year of birth: __ __ __ __

Year graduated medical school: __ __ __ __

How many years have you had your private clinic: _____ years

Video Observed Therapy Demo

Ethicality: *The extent to which the intervention has a good fit with an individual's value system*

1. Observing a patient taking his/her medicine is the best strategy for ensuring TB treatment adherence and completion.

[ ]	[ ]	[ ]	[ ]	[ ]	[ ]
Strongly disagree ↓	Disagree ↓	Neither disagree nor agree	Agree	Strongly agree	Refuse

1a. If the above question = Strongly disagree or disagree: What do you feel is the best strategy for ensuring TB treatment adherence and completion?

2. I am willing to test new treatment approaches to help my TB patients regain their health.

[ ]	[ ]	[ ]	[ ]	[ ]	[ ]
Strongly disagree	Disagree	Neither disagree nor agree	Agree	Strongly agree	Refuse

Intervention Coherence: *The extent to which the participant understands the intervention and how it works*

3. By reviewing videos each day, I would be able to identify and address any medication side effects more quickly than the routine care provided in my private clinic.

[ ]	[ ]	[ ]	[ ]	[ ]	[ ]
Strongly disagree	Disagree	Neither disagree nor agree	Agree	Strongly agree	Refuse

4. By reviewing videos each day, I would be able to identify which TB patients were at risk of stopping their TB treatment more quickly than the routine care provided in my clinic.

[ ]	[ ]	[ ]	[ ]	[ ]	[ ]
Strongly disagree	Disagree	Neither disagree nor agree	Agree	Strongly agree	Refuse

Burden: *The perceived amount of effort that is required to participate in the intervention*

5. The time commitment required of **me** to review videos of my TB patients every day would be…

[ ]	[ ]	[ ]	[ ]	[ ]	[ ]
Very difficult	Difficult	Neither difficult nor easy	Easy	Very Easy	Refuse

6. The time commitment required of **my TB patients** to make and upload videos every day would be…

[ ]	[ ]	[ ]	[ ]	[ ]	[ ]
Very difficult	Difficult	Neither difficult nor easy	Easy	Very Easy	Refuse

Private Provider Acceptability Survey in English

Opportunity Cost: *The extent to which benefits, profits or values must be given up to engage in the intervention*

7. Using a Video Observed Therapy (VOT) platform would save me **time** compared to the routine follow up in my private clinic.

[ ]	[ ]	[ ]	[ ]	[ ]	[ ]
Strongly disagree	Disagree	Neither disagree nor agree	Agree	Strongly agree	Refuse

8. Using a Video Observed Therapy (VOT) platform would save me **money** compared to the routine follow up in my private clinic.

[ ]	[ ]	[ ]	[ ]	[ ]	[ ]
Strongly disagree	Disagree	Neither disagree nor agree	Agree	Strongly agree	Refuse

Perceived Effectiveness: *The extent to which the intervention is perceived as likely to achieve its purpose*

9. Monitoring TB treatment progress via an online Video Observed Therapy (VOT) platform would allow me to provide individualized, tailored care to my TB patients.

[ ]	[ ]	[ ]	[ ]	[ ]	[ ]
Strongly disagree	Disagree	Neither disagree nor agree	Agree	Strongly agree	Refuse

10. Using a Video Observed Therapy (VOT) platform would help more of my patients adhere to and complete their TB treatment.

[ ]	[ ]	[ ]	[ ]	[ ]	[ ]
Strongly disagree	Disagree	Neither disagree nor agree	Agree	Strongly agree	Refuse

Self-Efficacy: *The participant's confidence that they can perform the behavior(s) required to participate in the intervention*

11. I feel confident about my ability to regularly monitor the treatment adherence and progress of my TB patients using a Video Observed Therapy (VOT) platform.

[ ]	[ ]	[ ]	[ ]	[ ]	[ ]
Strongly disagree	Disagree	Neither disagree nor agree	Agree	Strongly agree	Refuse

12. I feel confident about my ability to differentiate the care I provide to my TB patients based on information collected from a Video Observed Therapy (VOT) platform.

[ ]	[ ]	[ ]	[ ]	[ ]	[ ]
Strongly disagree	Disagree	Neither disagree nor agree	Agree	Strongly agree	Refuse

Implementation / Usability

13. I am worried that a Video Observed Therapy (VOT) platform would not be able to keep my patients' information strictly confidential.

[ ]	[ ]	[ ]	[ ]	[ ]	[ ]
Strongly disagree	Disagree	Neither disagree nor agree	Agree	Strongly agree	Refuse

14. I would feel comfortable having a dedicated staff member (a nurse or health worker) review videos each day and assist me with communicating with my TB patients regarding their side effects and treatment adherence.

[ ]	[ ]	[ ]	[ ]	[ ]	[ ]
Strongly disagree ↓	Disagree ↓	Neither disagree nor agree	Agree	Strongly agree	Refuse

14a. If the above question = Strongly disagree or disagree – Why do you not feel comfortable with a dedicated staff member assisting you with video reviews and communication?

15. I would feel comfortable sharing data about TB treatment adherence from a Video Observed Therapy (VOT) platform with the National TB Program.

[ ]	[ ]	[ ]	[ ]	[ ]	[ ]
Strongly disagree	Disagree	Neither disagree nor agree	Agree	Strongly agree	Refuse

Affective Attitude: *How an individual feels about the intervention*

16. Video Observed Therapy (VOT) addresses a problem which my patients face.

[ ]	[ ]	[ ]	[ ]	[ ]	[ ]
Strongly disagree	Disagree	Neither disagree nor agree	Agree	Strongly agree	Refuse

17. I believe that using a Video Observed Therapy (VOT) platform would be beneficial to my private practice and to my patients.

[ ]	[ ]	[ ]	[ ]	[ ]	[ ]
Strongly disagree	Disagree	Neither disagree nor agree	Agree	Strongly agree	Refuse

18. Video Observed Therapy (VOT) would be relevant to all the TB patients treated in my private clinic.

[ ]	[ ]	[ ]	[ ]	[ ]	[ ]
Strongly disagree ↓	Disagree ↓	Neither disagree nor agree	Agree	Strongly agree	Refuse

18a. If the above question = Strongly disagree or disagree – For which patients is this treatment support approach most applicable?

19. If given the opportunity, I would use a VOT platform to help manage my patients

[ ] Yes [ ] No [ ] Refuse

Private Provider Practice

20. In the past month, have you started anyone on treatment for drug-sensitive TB at your private practice?

[ ] Yes [ ] No [ ] Refuse

20a. If yes, how many TB patients? _______

21. In the past month, have you started anyone on treatment for MDR-TB at your private practice?

[ ] Yes [ ] No [ ] Refuse

22. Do you currently host clinic hours in a government facility?

[ ] Yes [ ] No [ ] Refuse

22a. If yes to the above question, in which type government facility do you work?
[ ] City-level (such as PNT or 115 People's Hospital) [ ] District-level (Such as DGH or DTU) [ ] Ward-level (such as CHS) [ ] Military health facility
[ ] Other: ____

23. How did you learn about TB treatment practices?
[ ] Medical school [ ] Colleagues [ ] Internet [ ] Gov't guidelines
[ ] PNT hospital [ ] Other: ____

24. What information sources do you use to keep up-to-date on TB diagnosis and treatment?
[ ] Books [ ] Internet [ ] Scientific journal [ ] Continuing medical education [ ] Pharmaceutical reps
[ ] Other:____

25. For an average suspect which method(s) do you primarily use to diagnose TB? *Check all that apply*
[ ] Clinical evaluation [ ] Chest X-ray [ ] Smear microscopy [ ] Antibiotic Trial [ ] Mantoux/TST
[ ] Xpert MTB/RIF [ ] Culture [ ] Serological test (IGRA) [ ] CBC or ESR
[ ] Other:____

26. Write a prescription for a previously untreated adult case of sputum-positive, drug-sensitive, pulmonary tuberculosis weighing 50 Kg (110 lbs).

Drugs	Dosage	Duration

27. Ensuring my TB patients adhere to and complete treatment is a challenge in my private clinic.
[ ] Strongly disagree [ ] Disagree [ ] Neither disagree nor agree [ ] Agree [ ] Strongly agree [ ] Refuse

28. When you start someone on TB treatment, which type of follow up is most common for your average patient for the first two months (intensive phase)?
[ ] Patient takes medicines at home with daily check ins from me
[ ] Patient takes medicines at home with weekly check ins from me
[ ] Patient takes medicines at home with monthly check ins from me
[ ] Patient takes medicines at home with no supervision

29. If a TB patient stops visiting you before his/her treatment is complete, what do you or your staff currently do to encourage them to comeback?
[ ] Phone call [ ] Home visit [ ] Nothing [ ] Refuse
[ ] Other:____
