# Supplementary material for: Assessing private provider perceptions and the acceptability of video observed treatment technology for tuberculosis treatment adherence in three cities across Viet Nam
Source: PLoS One. 2021 May 7;16(5):e0250644. doi: 10.1371/journal.pone.0250644 (PMC8104441; doi:10.1371/journal.pone.0250644)
Supplement: S2 Survey — (PDF) [file pone.0250644.s009.pdf]

Private Provider Acceptability Survey in Vietnamese

Ngày khảo sát: __ __ - __ __ - __ __ __ __ **Họ tên phỏng vấn viên:** ________________________________

Họ tên bác sĩ: ___________________________ **Giới tính:** [ ] Nam [ ] Nữ **Quận/Phường:** ____________

Năm sinh: __ __ __ __ **Năm tốt nghiệp ĐH Y:** __ __ __ __ **Thời gian hoạt động của phòng khám của BS:** _____ năm

Giới thiệu Ứng dụng ghi hình trong giám sát điều trị

Đạo đức: *Đánh giá mức độ sự phù hợp của can thiệp này với những giá trị đạo đức cá nhân*

1. Quan sát bệnh nhân uống thuốc là chiến lược tốt nhất để đảm bảo sự tuân thủ và hoàn thành điều trị lao.

[ ]	[ ]	[ ]	[ ]	[ ]	[ ]
Rất không đồng ý ↓	Không đồng ý ↓	Bình thường	Đồng ý	Rất đồng ý	Từ chối

1a. Nếu Câu trên= Rất không đồng ý hay Không đồng ý– BS cảm thấy chiến lược nào là tốt nhất để đảm bảo sự tuân thủ và hoàn thành điều trị lao?

2. BS sẵn sàng thử nghiệm các cách tiếp cận điều trị mới để giúp bệnh nhân hồi phục sức khỏe.

[ ]	[ ]	[ ]	[ ]	[ ]	[ ]
Rất không đồng ý	Không đồng ý	Bình thường	Đồng ý	Rất đồng ý	Từ chối

Tính chặt chẽ của can thiệp: *Mức độ BS hiểu về can thiệp và cách thức can thiệp được thực hiện*

3. Dùng clip của bệnh nhân gửi hàng ngày, BS sẽ có thể phát hiện và xử trí các tác dụng phụ, biến cố bất lợi nhanh hơn so với các bệnh nhân đến khám ở phòng khám của BS theo thường lệ.

[ ]	[ ]	[ ]	[ ]	[ ]	[ ]
Rất không đồng ý	Không đồng ý	Bình thường	Đồng ý	Rất đồng ý	Từ chối

4. Dùng clip của bệnh nhân gửi hàng ngày, BS sẽ có thể phát hiện bệnh nhân có nguy cơ bỏ trị nhanh hơn so với việc bệnh nhân đến khám ở phòng khám của BS theo thường lệ.

[ ]	[ ]	[ ]	[ ]	[ ]	[ ]
Rất không đồng ý	Không đồng ý	Bình thường	Đồng ý	Rất đồng ý	Từ chối

Công việc thêm: *Nhận thức về công sức cần cho việc tham gia vào can thiệp này*

5. BS cảm thấy việc sắp xếp thời gian để **BS** xem đoạn phim của bệnh nhân của mình hàng ngày …

[ ]	[ ]	[ ]	[ ]	[ ]	[ ]
Rất khó	Khó	Không khó không dễ	Dễ	Rất dễ	Từ chối

6. BS cảm thấy yêu cầu về thời gian để **bệnh nhân** quay phim và tải video mỗi ngày là …

[ ]	[ ]	[ ]	[ ]	[ ]	[ ]
Rất khó	Khó	Không khó không dễ	Dễ	Rất dễ	Từ chối

Chi phí cơ hội dự kiến: *Đánh giá mức độ lợi ích, lợi nhuận và giá trị bỏ ra khi tham gia vào can thiệp*

7. Sử dụng ứng dụng ghi hình trong giám sát điều trị (VOT) sẽ tiết kiệm **thời gian** của BS so với việc hẹn bệnh nhân đi tái khám nhiều lần tại phòng khám của BS.

[ ]	[ ]	[ ]	[ ]	[ ]	[ ]
Rất không đồng ý	Không đồng ý	Bình thường	Đồng ý	Rất đồng ý	Từ chối

8. Sử dụng ứng dụng ghi hình trong giám sát điều trị (VOT) sẽ tiết kiệm **chi phí** của BS so với việc hẹn bệnh nhân đi tái khám nhiều lần tại phòng khám của BS.

[ ]	[ ]	[ ]	[ ]	[ ]	[ ]
Rất không đồng ý	Không đồng ý	Bình thường	Đồng ý	Rất đồng ý	Từ chối

Hiệu quả dự kiến: *Đánh giá mức độ can thiệp sẽ đạt được hiệu quả như dự kiến*

9. Giám sát tiến trình điều trị của bệnh nhân qua ứng dụng trực tuyến ghi hình trong giám sát điều trị (VOT) sẽ cho phép bác sĩ có các biện pháp chăm sóc điều trị phù hợp cho từng bệnh nhân.

[ ]	[ ]	[ ]	[ ]	[ ]	[ ]
Rất không đồng ý	Không đồng ý	Bình thường	Đồng ý	Rất đồng ý	Từ chối

10. Sử dụng Ứng dụng ghi hình trong giám sát điều trị (VOT) sẽ giúp tăng số lượng bệnh nhân tuân thủ và hoàn thành điều trị lao.

[ ]	[ ]	[ ]	[ ]	[ ]	[ ]
Rất không đồng ý	Không đồng ý	Bình thường	Đồng ý	Rất đồng ý	Từ chối

Tự tin vào khả năng bản thân: *Người tham gia tự tin vào khả năng của bản thân rằng họ có thể thực hiện các hoạt động cần thiết để tham gia vào can thiệp này*

11. Bác sĩ tin là mình có thể theo dõi thường xuyên sự tuân thủ điều trị và sự tiến triển điều trị của bệnh nhân qua việc sử dụng ứng dụng ghi hình trong điều trị lao (VOT).

[ ]	[ ]	[ ]	[ ]	[ ]	[ ]
Rất không đồng ý	Không đồng ý	Bình thường	Đồng ý	Rất đồng ý	Từ chối

12. Bác sĩ tin là mình sẽ chăm sóc điều trị và cung cấp dịch vụ theo y học cá nhân cho bệnh nhân tốt hơn dựa trên thông tin thu thập từ Ứng dụng ghi hình trong giám sát điều trị.

[ ]	[ ]	[ ]	[ ]	[ ]	[ ]
Rất không đồng ý	Không đồng ý	Bình thường	Đồng ý	Rất đồng ý	Từ chối

Thực hiện / Khả năng sử dụng

13. BS lo lắng ứng dụng ghi hình trong giám sát điều trị lao (VOT) sẽ không giữ được bí mật thông tin bệnh nhân.

[ ]	[ ]	[ ]	[ ]	[ ]	[ ]
Rất không đồng ý	Không đồng ý	Bình thường	Đồng ý	Rất đồng ý	Từ chối

14. BS cảm thấy thoải mái khi có nhân viên nghiên cứu hỗ trợ (y tá hoặc nhân viên y tế) kiểm tra video mỗi ngày và hỗ trợ bác sĩ trong việc giao tiếp với bệnh nhân về việc tuân thủ điều trị và các tác dụng phụ.

[ ]	[ ]	[ ]	[ ]	[ ]	[ ]
Rất không đồng ý	Không đồng ý	Bình thường	Đồng ý	Rất đồng ý	Từ chối
↓	↓				

14a. Nếu câu hỏi trên = Rất không đồng ý hay Không đồng ý – Tại sao bác sĩ cảm thấy không thoải mái với việc nhân viên hỗ trợ mình trong việc kiểm tra video và giao tiếp với bệnh nhân?

15. BS có cảm thấy thoải mái khi chia sẻ dữ liệu về việc tuân thủ điều trị lao từ Ứng dụng ghi hình trong giám sát điều trị lao (VOT) với Chương trình chống lao quốc gia.

[ ]	[ ]	[ ]	[ ]	[ ]	[ ]
Rất không đồng ý	Không đồng ý	Bình thường	Đồng ý	Rất đồng ý	Từ chối

Thái độ cảm xúc: *Cá nhân cảm nhân như thế nào về can thiệp*

16. Ứng dụng ghi hình trong giám sát điều trị (VOT) giải quyết được vấn đề mà bệnh nhân đang đối mặt.

[ ]	[ ]	[ ]	[ ]	[ ]	[ ]
Rất không đồng ý	Không đồng ý	Bình thường	Đồng ý	Rất đồng ý	Từ chối

17. BS tin rằng sử dụng Ứng dụng ghi hình trong giám sát điều trị (VOT) là có lợi cho phòng khám của BS và bệnh nhân.

[ ]	[ ]	[ ]	[ ]	[ ]	[ ]
Rất không đồng ý	Không đồng ý	Bình thường	Đồng ý	Rất đồng ý	Từ chối

18. Ứng dụng ghi hình trong giám sát điều trị (VOT) có phù hợp với tất cả bệnh nhân được điều trị trong phòng khám của BS.

[ ]	[ ]	[ ]	[ ]	[ ]	[ ]
Rất không đồng ý	Không đồng ý	Bình thường	Đồng ý	Rất đồng ý	Từ chối
↓	↓				

18a. Nếu câu hỏi trên = Rất không đồng ý hay Không đồng ý – Phương pháp hỗ trợ điều trị này phù hợp với bệnh nhân nào?

19. Nếu có cơ hội, BS sẽ sử dụng ứng dụng VOT để quản lí bệnh nhân

[ ] Có [ ] Không [ ] Từ chối

Thực hành ở phòng khám của BS

20. Trong tháng qua, bác sĩ có bắt đầu điều trị cho bệnh nhân lao thông thường (nhạy cảm với thuốc lao hàng đầu) nào tại phòng khám?

[ ] Có [ ] Không [ ] Từ chối

20a. Nếu có, bao nhiêu bệnh nhân? ________

21. Trong tháng qua, bác sĩ có bắt đầu điều trị cho bệnh nhân lao đa kháng thuốc (kháng với thuốc rifampicin và isoniazid) tại phòng khám?

[ ] Có [ ] Không [ ] Từ chối

22. BS có hoạt động phòng khám trong cơ sở y tế công lập nào không?

[ ] Có [ ] Không [ ] Từ chối

22a. Nếu trả lời Có cho câu hỏi trước, BS làm việc cho cơ sở y tế công lập nào?

[ ] Tuyến thành phố (như BV Phạm Ngọc Thạch hay BVND115)	[ ] Tuyến quận (như Bệnh viện quận hay Tổ chống lao)	[ ] Tuyến phường (như trạm y tế xã)	[ ] Cơ sở y tế của quân đội

[ ] Khác: ____

23. BS học kiến thức về thực hành điều trị lao bằng cách nào?

[ ] Trường y khoa [ ] Đồng nghiệp [ ] Internet [ ] Hướng dẫn của Bộ y tế
[ ] BV Phạm Ngọc Thạch [ ] Khác: ____

24. BS đã sử dụng những nguồn thông tin nào để cập nhật kiến thức về chẩn đoán và điều trị bệnh lao?

[ ] Sách [ ] Internet [ ] Tạp chí khoa học [ ] Điều trị y khoa liên tục (CME) [ ] Trình dược viên
[ ] Khác:____

25. Đối với người nghi lao, BS sẽ chẩn đoán bệnh lao chủ yếu dựa trên phương pháp nào? <u>Chọn tất cả các lựa chọn phù hợp</u>

[ ] Đánh giá lâm sàng	[ ] X-quang ngực	[ ] Soi đờm	[ ] Tác động của kháng sinh phổ rộng	[ ] Mantoux/TST
[ ] Xpert MTB/RIF	[ ] Nuôi cấy	[ ] Xét nghiệm huyết thanh học (IGRA)	[ ] XN công thức máu (CBC), XN ESR	

[ ] Khác:____

26. Nhờ bác sĩ kê đơn thuốc cho một trường hợp bệnh nhân lao phổi người lớn nhạy cảm với thuốc, <u>chưa được điều trị trước đó</u> có kết quả đờm dương tính, nặng khoảng 50 Kg.

Thuốc	Liều	Thời gian điều trị

27. Đảm bảo bệnh nhân lao tuân thủ và hoàn thành điều trị là thách thức ở phòng khám của BS.

[ ]	[ ]	[ ]	[ ]	[ ]	[ ]
Rất không đồng ý	Không đồng ý	Bình thường	Đồng ý	Rất đồng ý	Từ chối

28. Khi BS kê đơn điều trị lao, BS thường sử dụng biện pháp theo dõi nào cho bệnh nhân lao trong 2 tháng đầu tiên (giai đoạn tấn công)

[ ] Bệnh nhân sử dụng thuốc tại nhà có sự kiểm tra hàng ngày của BS
[ ] Bệnh nhân sử dụng thuốc tại nhà có sự kiểm tra hàng tuần của BS
[ ] Bệnh nhân sử dụng thuốc tại nhà có sự kiểm tra hàng tháng của BS
[ ] Bệnh nhân sử dụng thuốc tại nhà không có sự kiểm tra của BS

29. Nếu bệnh nhân lao không đến tái khám ở phòng khám của BS trước khi hoàn thành điều trị, BS hay nhân viên của BS làm gì để động viên BN quay trở lại phòng khám?

[ ] Điện thoại [ ] Đến thăm nhà BN [ ] Không làm gì [ ] Từ chối
[ ] Khác:____
