# Supplementary material for: Assessing private provider perceptions and the acceptability of video observed treatment technology for tuberculosis treatment adherence in three cities across Viet Nam
Source: PLoS One. 2021 May 7;16(5):e0250644. doi: 10.1371/journal.pone.0250644 (PMC8104441; doi:10.1371/journal.pone.0250644)
Supplement: Attachment — Submitted filename: VOT - Response to Reviewers.docx [file pone.0250644.s010.docx]

19 March 2021

PLOS ONE Editorial Office

Dear Dr. Kevin Schwartzman,

We sincerely thank you for your consideration of the research article entitled “*Assessing private provider perceptions and the acceptability of video observed treatment technology for tuberculosis treatment adherence in three cities across Viet Nam*”.

We further thank you for your thorough review and thoughtful comments, and believe that by addressing the points raised we have been able to improve the quality and relevance of the manuscript. Below we have provided a point-by-point response (*italics*). Wherever appropriate, we have provided line numbers of the edits. These numbers correspond to those in the revised version showing all tracked changes. Text additions in full sentences have been underlined to improve readability of our responses.

**Reviewer 1 comments**

1. Please ensure that your manuscript meets PLOS ONE's style requirements, including those for file naming.

- *Thank you for including the PLOS ONE style templates. We have made changes to the manuscript, supporting information, and file naming conventions to ensure our formatting matches the requirements.*

2. Please include a copy of the questionnaire, in the original language, as Supporting Information, or include a citation if it has been published previously.

- *We have included the questionnaire in Vietnamese in the Supporting Information (S7 Survey).*

3. Please provide further details on sample size and power calculations.

- *The initial sample size calculation was based on a 2-sample, 2-sided comparison of mean scores on a 5-point Likert scale using the formula [1]: (1+1/κ)*(σ*(Z_1−α/2_+Z_1−β_)/(μ_A_−μ_B_))^2^. We chose α=95% and β=80% and applied a sampling ratio of κ=1. The difference in rating (μ_A_−μ_B_=d) between the bifurcated providers who would (μ_A_) and would not use VOT (μ_B_) was estimated to be 0.9 with a population variance (σ^2^) of 4. These values were estimated based on initial results from piloting the survey instrument and yielded a sample size of n=70. The final sample size of 79 represented an oversampling of 13% in case we had to exclude invalid participant responses post-hoc.*
- *Thus, to address your comment, we have added the following clarification in the Methods section: “The calculated sample size was 70 based on a 2-sample, 2-sided comparison of mean scores on a 5-point Likert scale with a 95% confidence level and 80% power. We estimated an average rating difference between private providers of 0.9 and a population variance of 4 based on initial results from piloting the survey instrument and intentionally oversampled for the eventuality of post-hoc exclusion of unclear or invalid responses.” (lines 202-206).*
- *However, given the opportunity we would like to also address the limitations in our sample size estimation and sampling methods.*
- *First, our target population (private providers treating tuberculosis patients) is difficult to identify. Due to limited evidence on private sector VOT perceptions in Viet Nam and other high-burden settings, we were unable to externally verify our estimates for the mean difference in provider responses and population variance.*
- *Second, private sector TB treatment in Viet Nam is highly restricted and providers are expected to refer TB patients to the public sector. Practicing in the private sector, especially for TB treatment, is sensitive and many private providers did not want to be identified. Lists of licensed private healthcare providers from the city’s regulatory authority do not identify private providers who treat tuberculosis patients in their private practice. We invited all eligible private providers treating tuberculosis in our network to complete the survey and employed a chain-sampling to approach as many eligible private providers as possible. Nevertheless, approaching this population proved to be a major challenge.*
- *Thus, we also added this as a limitation in the Discussion section: “Currently, there are no published data on private provider perceptions of VOT for TB treatment in Viet Nam or elsewhere, so we were unable to verify the validity of the mean difference and population variance estimates in our sample size calculation and therefore may have underpowered the study.” (lines 932-938).*

4. Please clarify how many hospitals are represented in this sample of 101 providers.

- *Within the 101 approached providers, all were operating at least a private practice. Our interviews were hence conducted at their private clinics or nearby locations at the private providers’ convenience. It is quite common in Viet Nam, however, that many private providers operate their practices to supplement a day job at a public hospital.*
- *As our study utilized our PPM network and chain-sampling, we were able to approach private providers with larger volumes of patients.*
- *Our survey provided the following information: in our sample of 101 private providers, 84 providers worked in both the private and public sectors. 85 private providers have their own clinics (formal or informal) at home. The sample of 101 private providers represents 85 single-doctor clinics and 8 private hospitals or polyclinics. Among 79 private providers interviewed, 65 providers worked in both the private and public sectors. Across 3 cities, 50 providers worked in city-level public hospitals (specialized tuberculosis hospital and general hospitals). There were 15 providers in HCMC working in military, district or ward-level hospitals.*
- *We have included this information in the Results section: “We invited 101 eligible private providers to participate, which included 85 single-doctor clinics and eight private hospitals or multi-clinics. Overall, 79 (78%) private providers agreed to participate in the study, with 65 of these providers working in both the private and public sectors.” (lines 289-291).*

5. In the Methods section, please clarify whether intra-cluster correlation was considered during estimation of the effective sample size - given that different localities were sampled. Further, in the Statistical analysis section, please elaborate how you accounted for clustering by locality in your statistical models. Did you consider multilevel models.

- *Thank you very much for the astute observation. We fully concur in retrospect and have adjusted the analysis to employ mixed-effect regressions with city as the random effect variable.*
- *As such, we have edited the methods section to reflect this change: “We fitted mixed ordinal proportional odds models [2] to explore the association between individual acceptability constructs and providers’ willingness to use VOT as the fixed effect variables, and city as the random effect. In the event of small or empty cells in individual groups, we aggregated individual 5-point Likert scale responses into three categories to stabilize the model: 1) Disagree, 2) Neither disagree nor agree, and 3) Agree. When the assumption of proportional odds was violated based on comparing* *models both assuming and relaxing the proportional odds assumption, we used mixed non-proportional odds models.” (lines 265-276).*
- *In the Results section, Table 3 (Constructs of acceptability associated with the willingness to use VOT in all three cities) and the text above it were edited to reflect the mixed ordinal regression model: “****Table 3*** *below shows that between private providers who would and would not use VOT, there are statistically significant differences in the majority of acceptability constructs. The three notable factors on which no significant difference was measured were the perceptions that VOT would help identify people at risk of stopping treatment faster (p=0.555), time requirement from doctors (p=0.413) and the doctors’ concerns about patient confidentiality (p=0.544). We have provided stratified analyses for HCMC (Table in S3 Table) and Ha Noi & Hai Phong (Table in S5 Table) in the Supporting information.” (lines 783-789).*

6. In statistical methods, please refer to any post-hoc corrections to correct for multiple comparisons during your statistical analyses. If these were not performed please justify the reasons. Please refer to our statistical reporting guidelines for assistance (<https://journals.plos.org/plosone/s/submission-guidelines.#loc-statistical-reporting>).
   - *Residual comparison was used as a post hoc test after a statistically significant chi-squared test [3]. We have edited the Methods section to reflect this change: “If these tests were statistically significant, residual comparison was used as a post-hoc test to identify main contributors to the differences” (lines 261-262).*
   - *In the Results section, we have added two asterisks (**) after the data points in Tables 1 & 2 that are the main contributors to the significant differences based on the residual comparison post-hoc test. Residual comparison tests showed that data points from Ho Chi Minh City and Hai Phong contributed the most to statistically significant differences.*
7. We note that you have indicated that data from this study are available upon request. PLOS only allows data to be available upon request if there are legal or ethical restrictions on sharing data publicly. For information on unacceptable data access restrictions, please see <http://journals.plos.org/plosone/s/data-availability#loc-unacceptable-data-access-restrictions>.
   - *We have edited and uploaded the minimal anonymized data in the Supplemental information as S8 Dataset.*
8. Please include captions for your Supporting Information files at the end of your manuscript, and update any in-text citations to match accordingly. Please see our Supporting Information guidelines for more information: [http://journals.plos.org/plosone/s/supporting-information](about:blank).
   - *We have added the captions (lines 1170-1181), updated in-text citations, and properly labeled the Supporting information files.*

**Additional Editor Comments**

**Major Comments**

1. The manuscript is generally well-written and clear, and provides relevant information to readers. However, there are some limitations that need to be addressed by the authors. The first of these relates to the study sample.  The sample size is fairly limited for a quantitative survey (79 respondents), and it is not entirely clear to what extent respondents are representative of those providing TB care within the private sector in the three cities.  For example, many respondents were recruited because of association with the NGO Friends for International Tuberculosis Relief.  It may be that physicians who are associated with this group may be more open to innovation and collaboration than other private sector physicians.  In the Results section, it would be appropriate to clarify how many of the participants had pre-existing relationships with this NGO.
   - *We appreciate that you recognize out the challenge of recruiting private sector stakeholders to elucidating public health insights, which to our understanding is ubiquitous. We have outlined the assumptions and innate limitations underlying the study’s sample size above, and would like to reiterate the paucity of existing evidence and difficulties in engaging and recruiting private providers.*
   - *As such, we fully concur that there is risk of bias in our results and have highlighted that limitation in the Discussion section, where we indicate that our study likely contains selection bias (lines 939-942) and social desirability bias from our chain-sampling method (lines 947-950).*
   - *To address your request for clarification on the number of participants with pre-existing relationships with the NGO Friends for International Tuberculosis Relief, we have added the following text to the Results section: “Across the three cities, 17 (22%) participants had pre-existing relationships with FIT: 8 (24%) in HCMC, 2 (11%) in Ha Noi and 7 (25%) in Hai Phong.” (lines 313-314).*
   - *First, we moved the statement “including those who may not be open to new technology, unknown to our network, or would not like others to know about their practice” to lines 945-947 for better cohesion.*
   - *Second, we added the following statement to lines 948-954: “As such, our results likely overestimate the acceptability of VOT and may not be representative of all private practitioners providing TB care in Viet Nam as a whole. Nevertheless, we were able to approach private providers with a large volume of patients, whom we consider the target population for VOT implementation. We believe that the ability to engage these high-volume private providers enhances the generalizability of these findings at least in the Vietnamese context, as these often also represent key opinion leaders in the local context.”*
2. In the Introduction, there is some conflation of directly observed treatment (DOT) with the WHO DOTS strategy, of which DOT is one component.  In addition, there is emerging a more nuanced understanding of and approach to treatment observation:  studies and meta-analyses have had some conflicting findings with respect to the benefits of DOT, while TB survivors and advocates have emphasized that “one size fits all” DOT may be neither necessary nor appropriate in all contexts.  It would be relevant to incorporate these elements into the Introduction section, and emphasize that video DOT may allow treatment support and supervision in a more individualized and differentiated manner.

- *We concur with your assessment and have modified the text as follows: “The country’s National TB Control Program (NTP) has successfully increased the number of TB cases notified, linked more individuals to treatment, and achieved high treatment success rates. The NTP attributes these achievements to robust nationwide coverage of treatment services that included quality-assured diagnostics and standardized treatment regimen with supervision and patient support as well as facility-based directly observed treatment (DOT) as per national treatment guidelines.” (lines 71-76).*
- *We also conducted an updated literature review on DOT and incorporated this information into the introduction: “While studies have found DOT can improve treatment outcomes [4], a more nuanced understanding of treatment observation has emerged in recent years. There are often conflicting views on whether DOT actually results in better treatment outcomes as compared to unsupervised, self-administered treatment. In an attempt to reproduce previous findings on the impact of DOT, McKay et al. found a small, non-statistically significant difference between DOT and self-administered treatment, along with a lack of high-quality evidence to compare these TB treatment methods in the form of prospective cohort or randomized trials [5]. A recent patient costing survey in Viet Nam showed that 63% of people being treated for drug-susceptible TB experienced catastrophic costs (>20% of annual household income spent on the patient’s episode of TB), and the majority of out-of-pocket spending occurred during their TB treatment [6]. In recent years, WHO has endorsed a more “patient-centered approach to TB care” and recommended that TB interventions explore other treatment administration options such as digital technologies to provide more individualized treatment support [7,8]” (lines 77-89).*
- *Lastly, throughout the manuscript we have edited all references to DOTS, when the intent was to refer to DOT, e.g., lines 117, 118, 126, etc.*

3. Although it cannot be remedied at this time, the inclusion of qualitative data would have strengthened the study substantially, through further explanation of private health provider perspectives on why they might/might not be interested in adopting video DOT.  This should be mentioned as a limitation in the discussion section, and as a next step for future investigation.
   - *We agree that a qualitative component would add valuable insight and have included this suggestion in the discussion: “Finally, we believe that the inclusion of a qualitative component would have strengthened our study. Although we included a space in several of our questions for write-in explanations, these were seldom utilized. Future studies could benefit from conducting a qualitative study to better understand specific causes of provider attitudes towards VOT” (lines 955-962).*
4. It is unclear what the stratified analysis involving Ho Chi Minh City (page 13 and Table 3b) adds to the manuscript.  I would suggest removing the text on lines 224-229 and Table 3b.  This could be moved to the appendix if the authors wish.
   - *We agree with this suggestion and have moved table 3b to the Supporting information (S3 Table). We have also removed the text referencing the table from the main manuscript.*

**Minor Comments**

1. In the tables, please round all percentages to the nearest whole number.  Two decimal places are unnecessary.
   - *All percentages have been rounded to the nearest whole number.*

**Reviewer comments to the author**

1. A brief, but useful/appropriate intro (background on TB, DOTs etc) and reasons for carrying out research. However, though the authors chose to focus on private providers, they don't really go into reasons for this. Is the public sector already using VOT? Would it produce different findings (i.e. would VOT be more acceptable)? Or did the authors just think this is a good way of involving private practice?
   - *We discussed our main reasons for focusing on the private sector in the Introduction section, lines 164-177. This includes the popularity of choosing private sector treatment for TB, and the low rates of treatment completion in the private sector of Viet Nam.*
   - *We have also clarified our focus on the private sector by discussing the successful use of VOT in Viet Nam’s public sector in the Introduction:* “*One study on VOT was conducted in Viet Nam with patients from NTP clinics in Hanoi. It utilized an asynchronous platform (SureAdhere) and showed that VOT was feasible and resulted in high treatment adherence in a resource-limited setting [9]” (lines 157-160).*
2. Fair description of the research - sampling, chosen methods, data collection and analysis all described and discussed. Their literature search missed a couple of important papers – most likely as this research was carried out before publication?
   - *Thank you for bringing new papers to our attention; we have updated our literature review and included these studies in our Introduction: “Recent studies comparing DOT to VOT have found the latter to be more effective (achieves higher treatment completion rates) and acceptable (achieves higher patient satisfaction) as a treatment observation tool for both active and latent TB* [10–12]*.*

*VOT implementation in various countries has been shown to be acceptable and feasible in programmatic settings [13,14] and patients enrolled in VOT have similar treatment outcomes compared to those enrolled on DOT [9,12,15]” (lines 134-139).*

- *Alongside this comment, we have also included an updated assessment of the potential value of VOT in light of the ongoing SARS-CoV-2 pandemic: “Lastly, acceptability is a dynamic construct subject to current events. Like many other industries, our ongoing private sector engagement has shown that private provider businesses, particularly those treating TB and respiratory illnesses, have suffered heavily from the government’s rigorous contact tracing, social distancing and forced quarantine efforts due to the SARS-CoV-2 pandemic. While these efforts have led to Viet Nam’s relative success in managing SARS-CoV-2, the deleterious economic impact may have also changed perceptions among private providers to be more accepting of remote care and telemedicine solutions such as VOT. This may particularly be the case in outbreak areas such as Da Nang in July 2020, when patient consultation and follow-up was provided via mobile chat applications during community-wide shut downs” (lines 902-911).*

3. The instrument they devised for responses appears reasonable and clearly demonstrated some internal validity - It may be worth justifying the development of this tool in the absence of any internationally validated instrument.
   - *We have included this justification for the development of our survey in the Methods section: “We chose to develop our own survey due to the absence of any internationally validated instrument on the acceptability of mobile health technologies for TB.” (lines 222-224).*
4. Somewhat limited description of what VOT is and the type of VOT proposed in the study (using the sureadhere app, so asynchronous). I was able to follow, but not sure I could have if I didn't know about the sureadhere app.
   - *We have added more information on VOT in general to the Introduction: “The asynchronous method of treatment management allows patients to record and transmit a video of themselves ingesting their medication to a central platform. These videos are later reviewed by healthcare workers” (lines 127-129).*
   - *We also clarified that SureAdhere is an asynchronous application in the Methods: “Prior to fielding the survey, participants were introduced to the SureAdhere VOT platform (San Diego, USA), an asynchronous smartphone application, and were shown a 2-minute video demonstrating how VOT is used by patients (*[*https://youtu.be/DiE-3a1GTn0)*](https://youtu.be/DiE-3a1GTn0))*.” (lines 249-251).*
5. Gaining the opinion of providers/services on the use of VOT is important, and, as the authors say, there is a dearth of research in this area of. Description of the findings/results, is fairly specific to private healthcare in Vietnam. It would be helpful to comment that the findings, while not directly applicable, are relevant to other high burden settings.
   - *Thank you for this suggestion. We have included the following comment in the Discussion section: “The findings from our study are relatively specific to private health care in Viet Nam; however, this information is still relevant to other high TB burden countries with a large proportion of patients seeking care in the private sector [16]” (lines 964-967).*

Thank you very much once again for your review and assessment of our manuscript and please do not hesitate to contact us if there are any additional requests.

Sincerely and on behalf of the study team,

Luan Nguyen Quang Vo

Chairman, Friends for International TB Relief

5^th^ Floor, 68B Nguyen Van Troi, 8, Phu Nhuan, Ho Chi Minh City, Viet Nam

+84-902908004

[luan.vo@tbhelp.org](mailto:luan.vo@tbhelp.org)

**References**

1.	Chow S, Shao J, Wang H. Sample Size Calculations in Clinical Research. In: 2nd Ed. 2008. p. 58. (Chapman & Hall/CRC Biostatistics Series).

2.	Hedeker D. Methods for Multilevel Ordinal Data in Prevention Research. Prev Sci Off J Soc Prev Res. 2015 Oct;16(7):997–1006.

3.	Sharpe D. Your chi-square test is statistically significant: Now What? Pract Assess Res Eval. 2015 Jan 1;20:1–10.

4.	Zhang H, Ehiri J, Yang H, Tang S, Li Y. Impact of Community-Based DOT on Tuberculosis Treatment Outcomes: A Systematic Review and Meta-Analysis. PloS One. 2016 Feb 5;11:e0147744.

5.	McKay B, Castellanos M, Ebell M, Whalen CC, Handel A. An attempt to reproduce a previous meta-analysis and a new analysis regarding the impact of directly observed therapy on tuberculosis treatment outcomes. PLOS ONE. 2019 May 23;14(5):e0217219.

6.	Nhung NV, Hoa NB, Anh NT, Anh LTN, Siroka A, Lönnroth K, et al. Measuring catastrophic costs due to tuberculosis in Viet Nam. Int J Tuberc Lung Dis Off J Int Union Tuberc Lung Dis. 2018 01;22(9):983–90.

7.	World Health Organization. A patient-centred approach to TB care. World Health Organization. 2018;

8.	World Health Organization. Guidelines for treatment of drug-susceptible tuberculosis and patient care: 2017 update. 2017.

9.	Nguyen TA, Pham MT, Nguyen TL, Nguyen VN, Pham DC, Nguyen BH, et al. Video Directly Observed Therapy to support adherence with treatment for tuberculosis in Vietnam: A prospective cohort study. Int J Infect Dis IJID Off Publ Int Soc Infect Dis. 2017 Dec;65:85–9.

10.	Story A, Aldridge RW, Smith CM, Garber E, Hall J, Ferenando G, et al. Smartphone-enabled video-observed versus directly observed treatment for tuberculosis: a multicentre, analyst-blinded, randomised, controlled superiority trial. The Lancet. 2019 Mar 23;393(10177):1216–24.

11.	Chen S-H, Wang I, Hsu H-L, Huang C-C, Liu Y-J, Putri DU, et al. Advantage in privacy protection by using synchronous video observed treatment enhances treatment adherence among patients with latent tuberculosis infection. J Infect Public Health. 2020 Sep 1;13(9):1354–9.

12.	Garfein RS, Doshi RP. Synchronous and asynchronous video observed therapy (VOT) for tuberculosis treatment adherence monitoring and support. J Clin Tuberc Mycobact Dis. 2019 Dec 1;17:100098.

13.	Sekandi JN, Buregyeya E, Zalwango S, Dobbin KK, Atuyambe L, Nakkonde D, et al. Video directly observed therapy for supporting and monitoring adherence to tuberculosis treatment in Uganda: a pilot cohort study. ERJ Open Res [Internet]. 2020 Apr 6 [cited 2020 Jul 29];6(1). Available from: https://www.ncbi.nlm.nih.gov/pmc/articles/PMC7132038/

14.	Kumar AA, Costa AD, Das A, Srinivasa GA, D’Souza G, Rodrigues R. Mobile Health for Tuberculosis Management in South India: Is Video-Based Directly Observed Treatment an Acceptable Alternative? JMIR MHealth UHealth. 2019 Apr 3;7(4):e11687.

15.	Holzman SB, Zenilman A, Shah M. Advancing Patient-Centered Care in Tuberculosis Management: A Mixed-Methods Appraisal of Video Directly Observed Therapy. Open Forum Infect Dis [Internet]. 2018 Apr 1 [cited 2019 Apr 18];5(4). Available from: https://academic.oup.com/ofid/article/doi/10.1093/ofid/ofy046/4924819

16.	Stallworthy G, Dias HM, Pai M. Quality of tuberculosis care in the private health sector. J Clin Tuberc Mycobact Dis. 2020 Aug 1;20:100171.
