# Supplementary material for: Assessing private provider perceptions and the acceptability of video observed treatment technology for tuberculosis treatment adherence in three cities across Viet Nam
Source: PLoS One. 2021 May 7;16(5):e0250644. doi: 10.1371/journal.pone.0250644 (PMC8104441; doi:10.1371/journal.pone.0250644)
Supplement: Attachment — Submitted filename: Response to Reviewers2 - VOT.docx [file pone.0250644.s011.docx]

1 April 2021

PLOS ONE Editorial Office

Dear Dr. Kevin Schwartzman,

We sincerely thank you for your consideration of the research article entitled “*Assessing private provider perceptions and the acceptability of video observed treatment technology for tuberculosis treatment adherence in three cities across Viet Nam*”.

We further thank you for your review and comments, and believe that by addressing the points raised we have been able to improve the quality and relevance of the manuscript. Below we have provided a point-by-point response (*italics*). Wherever appropriate, we have provided line numbers of the edits. These numbers correspond to those in the revised version showing all tracked changes.

**Editor comments**

1. The first of these is your approach to multiple comparisons, which was raised by journal review staff. While I appreciate the use of the residual analysis, I think a more appropriate approach would be to correct directly for multiple comparisons (e.g. Bonferroni). In table 2, the repeated use of "**" to label variables deemed most responsible for differences between cities doesn't add much, as nearly all variables are labeled as such.
   - *Thank you very much for this suggestion. We have completed an additional review of this topic and chose to use the Holm-Bonferroni sequential correction [1] to account for multiple comparisons.* *The Holm-Bonferroni sequential correction is generally more powerful and less conservative than the classical Bonferroni test [2]*. *As such, we have edited the Methods section to reflect this change:* *“If these tests were statistically significant, the Holm-Bonferroni sequential correction was applied to account for multiple comparisons” (lines 174-175).*
   - *In the Results section, Table 1 (Characteristics of private providers in the survey stratified by city) and Table 2 (Overall attitudes of private providers towards VOT) were edited to reflect the Holm-Bonferroni correction. We have removed the two asterisks “**” and instead used the symbol “^$^” to denote the acceptability constructs that retained statistical significance after the Holm-Bonferroni correction. We also added the following information “Detailed results using the Holm-Bonferroni sequential correction are provided in the supplemental information (Table S8)” (lines 235-237).*
2. A more minor point is that in tables 1 and 2, the rightmost column is labeled "chi-square test." In fact, as before this column displays P-values in both tables, and should be labeled as such i.e. "P-value." [In fact, in table 2, many of the P-values reflect the Fisher exact test rather than the chi-square test].
   - *Thank you for this comment. We have updated the label of the rightmost column of Tables 1 and 2 as "P-value”.*

Thank you very much once again for your review and assessment of our manuscript and please do not hesitate to contact us if there are any additional requests.

Sincerely and on behalf of the study team,

Luan Nguyen Quang Vo

Chairman, Friends for International TB Relief

5^th^ Floor, 68B Nguyen Van Troi, 8, Phu Nhuan, Ho Chi Minh City, Viet Nam

+84-902908004

[luan.vo@tbhelp.org](mailto:luan.vo@tbhelp.org)

**References**

1.	Holm S. A Simple Sequentially Rejective Multiple Test Procedure. Scand J Stat. 1979;6(2):65–70.

2.	What is the proper way to apply the multiple comparison test? [Internet]. [cited 2021 Mar 31]. Available from: https://ekja.org/journal/view.php?doi=10.4097/kja.d.18.00242
